# Executive Dysfunction in Autism Spectrum Disorder Is Associated With Increased Cerebro‐Cerebellar Resting‐State Functional Connectivity

**DOI:** 10.1155/np/7449692

**Published:** 2026-03-16

**Authors:** Xiaotong Zhang, Guorong Qiu, Zhiwei Mou, Changfu Chen, Jianliang Lu, Zhiming Tang, Zhaowen Zhou

**Affiliations:** ^1^ Department of Rehabilitation Medicine, The First People′s Hospital of Foshan (Foshan Hospital Affiliated to Southern University of Science and Technology), School of Medicine, Southern University of Science and Technology, Guangdong, China, fshospital.org.cn; ^2^ Department of Rehabilitation Medicine, Guangzhou Red Cross Hospital of Jinan University, Guangzhou, Guangdong, China, jnu.edu.cn; ^3^ College of Information Science and Technology, Jinan University, Guangzhou, Guangdong, China, jnu.edu.cn

**Keywords:** autism spectrum disorders, cerebro-cerebellar connectivity, executive function, resting state functional magnetic resonance imaging (rs-MRI)

## Abstract

This study examined the correlation between the cerebellar functional status and the executive function (EF) scores and explored alterations of functional connectivity (FC) among autism spectrum disorder (ASD) individuals compared to typically developing (TD) subjects. Individuals from the Autism Brain Imaging Data Exchange II dataset (ABIDE II) with complete cerebellum scanning coverage and available Behavior Rating Inventory of EF (BRIEF) *t*‐scores were included, yielding a final sample of 71 ASD (age: 11.50 ± 2.77) and 149 TD (age:11.48 ± 1.60) individuals. Four cerebellar ROIs (left Lobule VI, left Crus I, right Crus I, and left VIIB) were defined from a meta‐analysis. We quantified cerebellar intrinsic activity using percent amplitude of fluctuation (PerAF) and assessed seed‐based FC within cerebro‐cerebellar circuits. We found no between‐group differences in PerAF across the four ROIs, and PerAF showed no significant association with global executive composite (GEC) scores within the ASD group. In contrast, FC analyses revealed predominantly increased cerebro‐cerebellar connectivity in ASD. Notably, stronger FC between left Lobule VI and right inferior frontal gyrus, pars opercularis (IFGoper), as well as between left Crus I and left IFGoper were positively correlated with BRIEF Shift scores, indicating that greater coupling within these loops was associated with poorer cognitive flexibility. These results suggest that EF impairment among ASD individuals might be reflected in part by alterations in functional interconnections within the cerebro‐cerebellar system. These findings help to understand the potential role of the cerebellum in EF among ASD individuals and might provide ideas for therapeutic interventions.

## 1. Introduction

Autism spectrum disorder (ASD) comprises a collection of neurodevelopmental diseases defined by deficits in communication and social interaction and repetitive and restrictive behaviors [1]. The World Health Organization estimated that worldwide about 1 in 100 children suffer from autism [2]. Although the core symptom of ASD is social dysfunction, atypical cognitive processes in ASD contribute to the expression of behavioral symptoms. Impairment of executive function (EF) is prevalent in ASD and strongly predicts symptom severity [3] in the areas of theory of mind, social communication [4, 5], and restricted and repetitive behavior patterns [6]. Also, more severe executive dysfunction was associated with lower quality of life [7]. EF is an umbrella term for the set of higher‐order cognitive abilities required for pursuing and achieving goals [8], such as controlling, organizing, and directing cognitive activity, behavioral activity, and emotional response [9]. A meta‐analysis showed that impaired EF was present and stable across multiple domains (e.g., planning, working memory, mental flexibility) of development in ASD compared with age‐ and IQ‐matched control groups [10]. Despite their clinical significance, the neural mechanisms underlying EF deficits in ASD remain poorly understood, hindering targeted interventions.

The cerebellum is a major region where neuropathology was first detected in ASD [11]. In populations with ASD, Purkinje cell numbers were reduced in all lobules and the posterior lobe of the vermis of the cerebellum [11], as well as in the deep cerebellar nuclei [12]. From the molecular perspective, lower parvalbumin mRNA levels in Purkinje cells [13], reduced antiapoptotic proteins, and increased proapoptotic proteins [14] were found in the cerebellum in ASD. Damage to the cerebellum at birth resulted in high scores on the modified checklist for autism in toddlers (M‐CHAT) and Vineland autism screening inventories with a risk ratio as high as 40 [15]. This evidence suggests that cerebellar abnormalities in ASD might be associated with symptoms.

Accumulating neuroimaging evidence consistently links cerebellar abnormalities to multiple functional domains in ASD. For example, during a sequential finger‐tapping task, children with ASD show absent activation in contralateral cerebellar lobules IV/V and reduced premotor‐cerebellar connectivity [16]. In tasks requiring theory of mind, reduced activation in Crus I and decreased cerebellum–medial prefrontal cortex connectivity are observed [17]. Developmentally, ASD is associated with increasing cerebellar‐striatal connectivity over time, opposite to typical patterns [18]. Age‐specific local connectivity alterations are also noted, with lower cerebellar regional homogeneity in children and adults but increased local connectivity in adolescents [19]. Resting‐state fMRI studies further reveal reduced contributions to the right posterior cerebellum [20] and attenuated connectivity between the cerebellar vermis and occipitotemporal regions [21] in ASD, with severity linked to cerebelloparietal hypoconnectivity. These findings underscore the cerebellum as a critical region whose aberrant connectivity may contribute to the pathophysiology of ASD.

Although the relationship between executive dysfunction and cerebro‐cerebellar connectivity in children with ASD remains incompletely understood, increasing evidence supports the role of the cerebellum in cognition. Cerebellar insult, as a strong risk factor for ASD, also affects a wide range of cognition [22, 23]. A resting‐state fMRI study in ASD reported underconnectivity in cerebellar circuits involved in cognitive and higher‐order operation [24]. There are increasing studies investigating cerebellar involvement in cognitive processing. Research on cerebellar cognitive organization shows that lobules VI, Crus I, and VII are involved in language, working memory, spatial tasks, emotion, and EF [25]. Xue et al. [26] proposed a three‐tiered hierarchical model of the cerebellum, which emphasizes the crucial role of the Crus I/II apex in higher‐order cognitive functions. [27] revealed that lobule VII and lobule VI were activated in cognitive tasks. However, the specific regions and neural circuits involved in cognitive processes in the cerebellum remain controversial.

Converging neuroimaging evidence from both healthy and clinical populations implicates cerebellar circuits in EF performance and dysfunction. In healthy adults, performing functional tasks activates the posterior cerebellum [14], with meta‐analysis highlighting bilateral Crus I, left lobules VI and VIIB during EF tasks [28]. Resting‐state studies show that Crus I/II are consistently engaged across core EF tasks such as *n*‐back, task‐switching, and stop‐signal paradigms [29]. Further studies revealed the specific engagement of the cerebellum during EF tasks. For example, cognitive flexibility evaluated by the rule‐retrieval task activates the right cerebellum concurrently with left‐lateralized frontoparietal regions [30]. Recent diffusion MRI findings provide a structural basis for these functional associations. Structurally, diffusion MRI links white matter integrity in the left cerebellum and bilateral fornix to better attention, EF, and memory performance, while microstructural integrity of the right Crus I/middle cerebellar peduncle correlates with improved language‐related EF [31].

Investigations into clinical populations with known EF deficits reinforce the role of the cerebellum. In children with attention‐deficit/hyperactivity disorder (ADHD), meta‐analyses of task‐based fMRI studies consistently report cerebellar alterations during EF tasks, manifesting as both hyperactivation (e.g., in the cerebellar tonsils during inhibition) [32] and hypoactivation (e.g., in left Crus I/II across EF domains) [33]. These divergent patterns suggest a dysregulated cerebellar contribution to fronto‐cerebellar circuits, potentially underlying core EF impairments in ADHD. However, research examining cerebellar contributions to EF in children with ASD remains limited, and whether cerebellar pathway abnormalities represent a neural mechanism for EF impairment in ASD is still unclear. Given EF is critical to the quality of life of patients with ASD, this study aims to explore cerebellar regions associated with EF and investigate the altered functional connectivity (FC) between the cerebellum and cerebrum in individuals with ASD.

## 2. Methods

### 2.1. Participants

**Figure 1:**
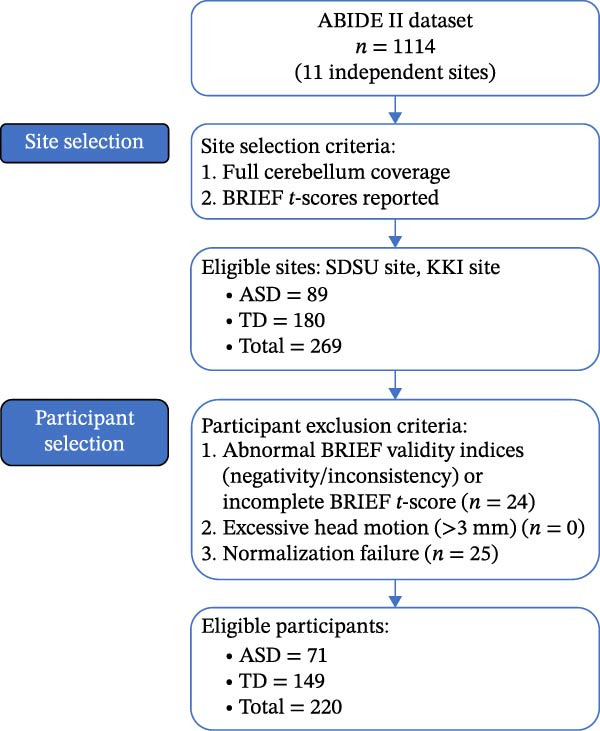
Flowchart of participant selection process.

Data used in the present study were obtained from the large‐scale Autism Brain Imaging Data Exchange II dataset (ABIDE II, https://fcon_1000.projects.nitrc.org/indi/abide/abide_II.html), which includes data from 1114 participants across 11 independent sites [34]. The whole dataset was downloaded, including all the fMRI data and the phenotypic data from all the participants. Imaging sites that provided scans covering the entire cerebellum and reported Behavior Rating Inventory of EF (BRIEF) *t*‐scores were selected. The imaging sites that met these criteria were the SDSU and KKI datasets (detailed MRI acquisition parameters for the two included sites as well as clinical characteristics of included ASD individuals for two sites are presented in Tables S1 and S2). Meanwhile, participants with BRIEF validity indices (i.e., negativity and inconsistency) outside the normal range or incomplete BRIEF *t*‐scores were excluded. Additionally, participants who exhibited excessive maximum head motion (>3 mm) or failure of normalization were excluded (Figure 1). Finally, 71 ASD and 149 typically developing (TD) individuals were included in the final analysis, the demographic data of the included individuals are shown in Table 1.

**Table 1:** Demographic data for individuals included in the present study.

Characteristic	ASD (*n* = 71)	TD (*n* = 149)	*Z*/*x* ^2^	*p*‐Value
Age	11.50 ± 2.77	11.48 ± 1.60	1.375	0.169
Sex	—	—	0.845	0.358
Male	52 (73.24%)	100 (67.11%)	—	—
Female	19 (26.76%)	49 (32.89%)	—	—
FIQ	102.55 ± 16.12	114.72 ± 10.46	5.497	<0.001
VIQ	106.04 ± 18.47	118.53 ± 11.93	5.152	<0.001
PIQ	104.13 ± 16.64	110.76 ± 12.10	3.145	0.002
BRIEF *t*‐score				
Inhibit	64.93 ± 13.10	43.75 ± 6.07	−10.273	<0.001
Shift	69.12 ± 11.66	43.27 ± 7.01	−10.939	<0.001
Emotional control	61.06 ± 12.06	43.27 ± 7.07	−9.410	<0.001
Initiate	64.85 ± 10.14	44.85 ± 7.66	−10.627	<0.001
Working memory	67.46 ± 10.68	44.26 ± 7.02	−10.894	<0.001
Plan/organize	67.27 ± 9.85	44.01 ± 7.86	−10.900	<0.001
Organization of materials	59.96 ± 11.28	47.61 ± 8.89	−7.187	<0.001
Monitor	65.49 ± 10.77	42.69 ± 8.64	−10.425	<0.001
BRI	66.77 ± 11.14	43.62 ± 6.41	−11.117	<0.001
MI	67.82 ± 9.38	43.62 ± 7.89	−11.133	<0.001
GEC	68.75 ± 9.38	42.77 ± 7.04	−11.396	<0.001

*Note:* FIQ, full IQ standard score; PIQ, performance IQ standard score; VIQ, verbal IQ standard score.

Abbreviations: BRI, Behavioral Regulation Index; BRIEF, Behavior Rating Inventory of Executive Function; GEC, global executive composite; MI, Metacognition Index.

### 2.2. Evaluation of Executive Function

The BRIEF Parent Form [35] is an 86‐item parent‐reported questionnaire used to assess EF of 5–18‐year‐old children. Each item is scored on a 3‐point Likert scale (1 = never, 2 = sometimes, 3 = often) according to the frequency with which children demonstrated EF difficulties. Higher scores indicate poorer EF performance. The Behavioral Regulation Index (BRI) consists of the Inhibit, Shift, and Emotional Control subscales that are all related to a child’s ability to use inhibitory control to shift cognitive set and to manage emotions and behavior. The Metacognition Index (MI) consists of the Initiate, Working Memory, Plan/Organize, Organization of Materials, and Monitor subscales, reflecting the ability to cognitively manage tasks and to monitor their performance. These indexes can be combined to calculate a general overall score, the global executive composite (GEC). Age‐ and sex‐specific norms were used to transform raw scores to *T*‐scores, which were used in our analyses. The BRIEF data were validated in terms of the validity scale scores (negativity and inconsistency).

### 2.3. Data Processing Strategy

In this study, we first examined whether cerebellar intrinsic activity (percent amplitude of fluctuation [PerAF]) differed between the ASD and TD groups. Mean PerAF values were extracted from four ROIs (5‐mm radius spheres): ROI1, left Lobule VI (−36, −66, −28); ROI2, left Crus I (−12, −78, −28); ROI3, right Crus I (30, −68, −34); and ROI4, left VIIB (−28, −78, −52). These ROIs were defined based on the cerebellum‐focused activation likelihood estimation (ALE) meta‐analysis, which identified convergent and reproducible cerebellar peak coordinates across functional neuroimaging studies related to EF in healthy participants. Within the ASD group, correlation analysis between PerAF values in these four ROIs with the *t*‐score of GEC was conducted. Second, we performed voxel‐wise FC analyses using the same four ROIs to identify cerebro‐cerebellar FC connectivity differences between individuals with ASD and TDs. Finally, the strength of connections showing significant between‐group differences was correlated with BRIEF *t*‐scores within the ASD group, hoping to find the potential correlation between the functional reorganization within the cerebro‐cerebellar loops and the EF.

### 2.4. Preprocessing

Restplus toolbox (Version 1.24, http://restfmri.net/forum/restplus) and SPM12 were used to perform the rs‐fMRI data analysis. All the rs‐fMRI data were first preprocessed using the following steps according to their corresponding parameters: (1) slice timing, (2) motion realignment, (3) spatial normalization into the MNI space, (4) spatial smoothing (with a 6‐mm FWHM Gaussian kernel), (5) removal of the linear time series, (6) regression of nuisance signals (Fristons’s 24 head motion, global mean signal, white matter signal, and cerebrospinal fluid signal), and (7) temporal bandpass filtering (0.01–0.08 Hz) across time series.

### 2.5. PerAF Analysis

In the present study, PerAF was used as the metric that quantifies the amplitude of spontaneous neural activity fluctuations. It measures the percentage of BOLD fluctuations relative to the average BOLD signal intensity at each time point and then averages these fluctuations over the entire time series [36]. PerAF is calculated by the following formula:

PerAF=1n∑i=1n Xi−μμ×100,

μ=1n∑i=1n Xi,

where *X*_*i*_ denotes the signal intensity at the *i*_th_ time point, *n* is the number of time points in the series, and *μ* represents the mean value of the entire time series [36]. PerAF was calculated using the Restplus toolbox.

To evaluate the correlation between PerAF and the *t*‐score of GEC, Spearman’s correlation analysis was used to explore the relationships between PerAF in each ROI and the *t*‐score of GEC. We controlled for multiple comparisons using the Benjamini–Hochberg FDR procedure across all tested correlations. FDR‐corrected *p*  < 0.05 was considered as statistically significant.

### 2.6. FC Analysis

For the FC analysis, the average time series from each ROI was extracted, and Pearson correlation coefficients were calculated between the ROI time series and the time series of each other voxel throughout the brain. To enhance normality, the FC maps were then transformed into zFC maps using Fisher’s r‐to‐z transformation. To compare the difference in FC between ASD patients and TDs, a voxel‐wise group comparison was performed with the use of an independent two‐sample *t*‐test. *p*  < 0.001 (FDR corrected, cluster size >30) was considered to be statistically significant.

In order to explore the relation of FC in altered cerebro‐cerebellar loops and EF among ASD individuals, Spearman’s correlation analysis was used to explore the correlation between the *t*‐score of BRIEF and zFC values. We controlled for multiple comparisons using the Benjamini–Hochberg FDR procedure across all tested correlations. FDR‐corrected *p*  < 0.05 was considered as statistically significant.

### 2.7. Statistical Analysis

Statistical analysis of the fMRI data was performed as discussed above. Analyses of demographic and clinical characteristics were conducted using SPSS 20.0. The distribution of the data were confirmed by the Shapiro–Wilk test and the Q–Q plots. Fisher’s exact test and Mann–Whitney *U* test were used to evaluate group differences. *p*  < 0.05 was considered as statistically significant.

## 3. Results

### 3.1. Demographic Results

In the present study, 71 ASD were included following the inclusion and exclusion criteria, and 149 age‐ and sex‐matched TD were also included. There were no significant differences in age and sex between the two groups (*p*  > 0.05). Demographic data are shown in Table 1.

### 3.2. PerAF Results

Between‐group comparison showed no significant differences in PerAF across four ROIs (Table 2). In addition, correlation analyses in the ASD group revealed no significant associations between PerAF values in any ROI and GEC after FDR correction. Detailed results are presented in Table 3.

**Table 2:** Group differences in PerAF between ASD and TD groups.

PerAF	ASD (Mean ± SD)	TD (Mean ± SD)	*t*	*p*	FDR‐adjusted *p*
ROI1	0.845 ± 0.257	0.768 ± 0.240	2.143	0.034	0.136
ROI2	0.656 ± 0.189	0.651 ± 0.160	0.184	0.854	0.854
ROI3	0.567 ± 0.132	0.557 ± 0.120	0.578	0.564	0.752
ROI4	0.914 ± 0.370	0.880 ± 0.322	0.675	0.501	0.752

**Table 3:** Correlations between PerAF in four ROIs and GEC in the ASD group.

PerAF	*r*	*p*	FDR‐adjusted *p*
ROI1	−0.025	0.836	0.836
ROI2	−0.082	0.496	0.661
ROI3	−0.247	0.038	0.153
ROI4	−0.147	0.222	0.443

### 3.3. FC Results

#### 3.3.1. FC Comparison Results

**Figure 2:**
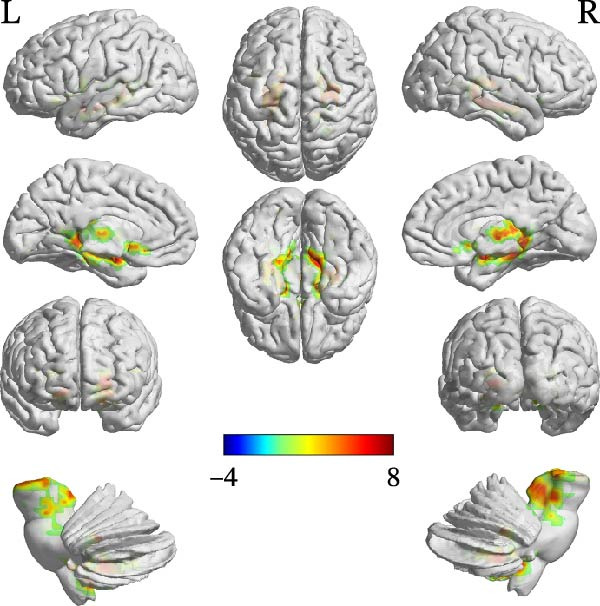
FC comparison results using ROI1 as seed region (ASD > TD).

**Figure 3:**
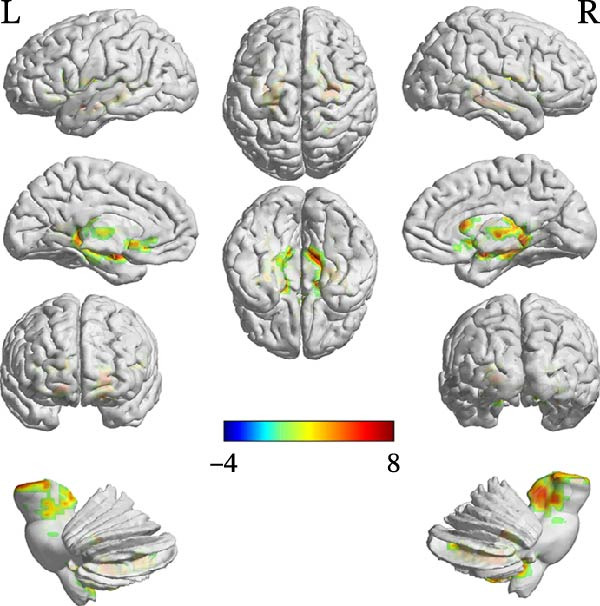
FC comparison results using ROI2 as seed region (ASD > TD).

**Figure 4:**
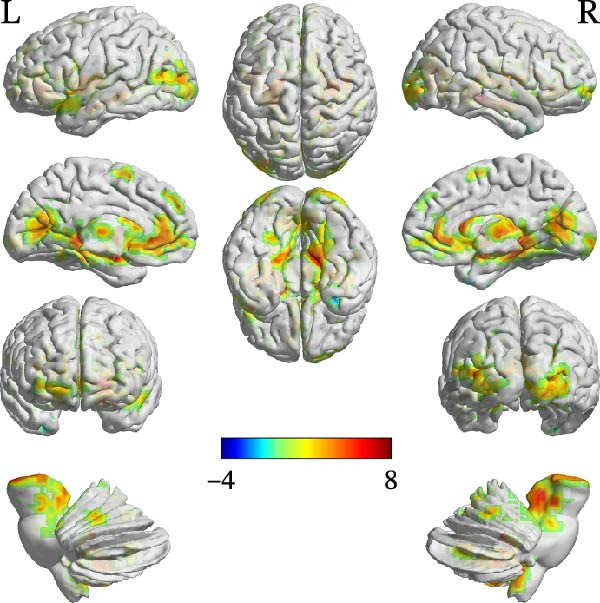
FC comparison results using ROI3 as seed region (ASD > TD).

**Figure 5:**
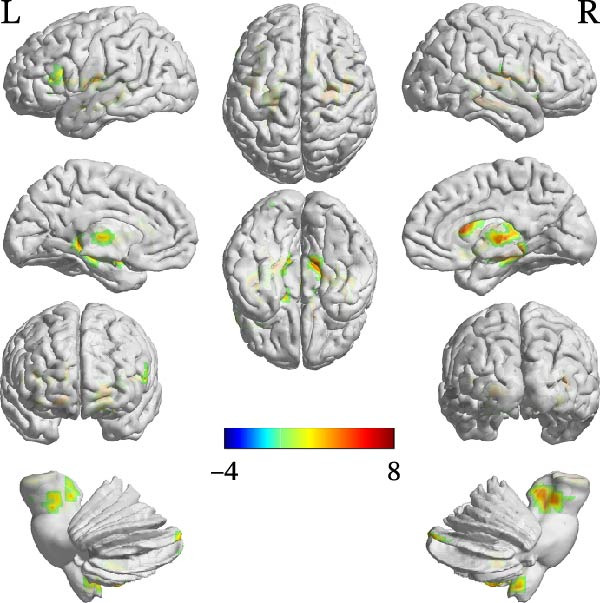
FC comparison results using ROI4 as seed region (ASD > TD).

Compared with the TD group, the ASD group showed increased FC between the cerebellar ROIs and a distributed set of regions, including subcortical areas (e.g., thalamus, hippocampus, and caudate), frontal areas (e.g., left inferior frontal gyrus, pars opercularis [IFGoper] and supplementary motor area), and temporal regions (e.g., fusiform gyrus and Heschl’s gyrus). Conversely, decreased FC was observed between the right Crus I and the right middle temporal pole. Detailed peak coordinates, cluster sizes, and statistics are presented in Table 4 and Figures 2–5 (between‐group difference in seed‐based FC analysis (ASD < TD) is presented in Table S3).

**Table 4:** Between‐group difference in seed‐based FC analysis (ASD > TD).

Region		MNI coordinates				
		*x*	*y*	*z*	Cluster size	Peak‐*t*
ROI1						
A	Left Lobule IX	−12	−39	−45	308	6.9396
B	Right thalamus	3	−12	0	1459	7.4043
C	Left fusiform gyrus	−21	−81	−6	30	4.2151
D	Left inferior frontal gyrus (pars opercularis)	−36	18	15	68	4.7588
E	Right inferior frontal gyrus (pars opercularis)	39	18	15	38	4.4017
ROI2						
A	Right Vermis_10	6	−51	−27	377	6.0601
B	Right hippocampus	30	−39	6	1449	7.384
C	Left inferior frontal gyrus (pars opercularis)	−39	15	15	120	5.2124
D	Right inferior frontal gyrus (pars triangularis)	45	24	12	143	4.8059
ROI3						
A	Right Vermis_10	6	−51	−27	659	6.6994
B	Right middle temporal pole	30	12	−42	35	−3.7896
C	Right hippocampus	24	−33	3	7801	6.6154
D	Right middle frontal gyrus	36	36	21	31	3.3462
E	Left medial superior frontal gyrus	0	54	33	37	3.4324
F	Left middle frontal gyrus	−30	12	39	31	4.2576
G	Left superior frontal gyrus	−12	6	51	79	4.1773
H	Left supplementary motor area	0	9	63	51	3.4455
ROI4						
A	Right Lobule IX	3	−57	−51	67	5.2089
B	Left Crus II	−21	−90	−30	36	4.5912
C	Right Crus I	33	−87	−30	63	4.7944
D	Right hippocampus	30	−39	6	212	5.3592
E	Left hippocampus	−24	−30	−6	227	5.0143
F	Right Heschl gyrus	42	−21	6	319	5.5171
G	Left inferior frontal gyrus (pars opercularis)	−36	15	15	146	5.2866
H	Right thalamus	3	−18	3	88	6.0136
I	Left rolandic operculum	−42	−12	12	99	4.4775
J	Right caudate	9	18	12	51	5.4608

#### 3.3.2. Correlation Results

Correlation analysis indicated that the *t*‐score of the shift subscale in BRIEF was positively correlated with FC strength within left Lobule VI–right IFGoper (*r* = 0.429, *p*  < 0.001, FDR‐adjusted *q* = 0.028) and left Crus I–left IFGoper (*r* = 0.431, *p*  < 0.001, FDR‐adjusted *q* = 0.028). All associations reported above survived Benjamini–Hochberg FDR correction across all tested correlations. Detailed results are provided in Figure 6. Complete correlation results are provided in the (Figures S1–S4).

Figure 6Correlation analysis results: (a) BRIEF shift T‐scores correlated with FC strength between left Lobule VI and right IFGoper (*r* = 0.429, *p* < 0.001, FDR‐adjusted *q* = 0.028) and (b) BRIEF shift T‐scores correlated with FC strength between left Crus I and left IFGoper (*r* = 0.431, *p* < 0.001, FDR‐adjusted *q* = 0.028).

**Figure:**
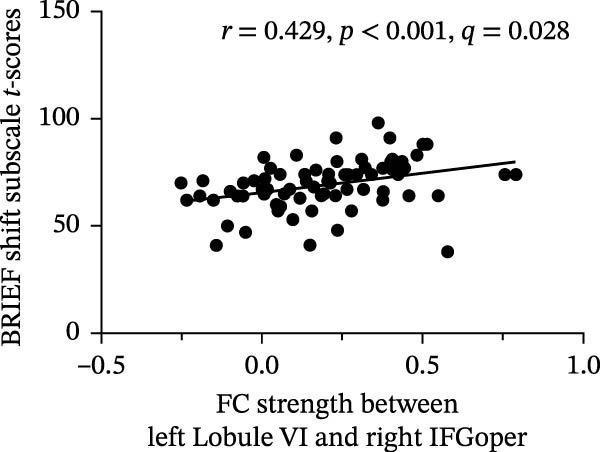
(a)

**Figure:**
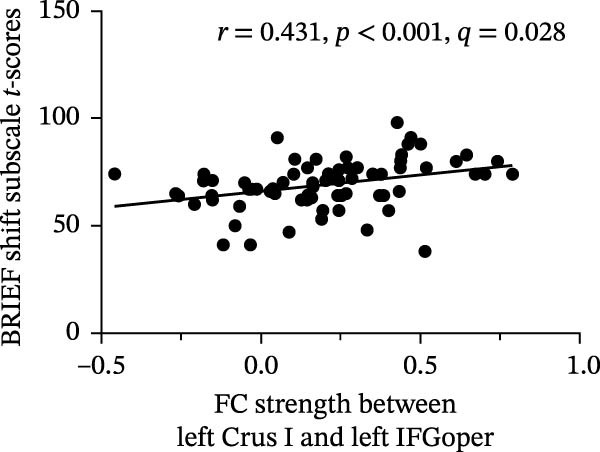
(b)

## 4. Discussion

In the present study, no significant between‐group differences in PerAF across the four ROIs were found after FDR correction. Similarly, no significant associations were found between PerAF and GEC scores within the ASD group. In contrast, FC analysis demonstrated predominantly increased FC in cerebro‐cerebellar circuits in the ASD individuals compared with TD participants. Notably, FC strength between the left Lobule VI and right IFGoper, as well as between the left Crus I and left IFGoper, was positively correlated with the shift subscale in BRIEF, indicating that higher FC within these cerebro‐cerebellar loops correlated with poorer executive control.

### 4.1. Cerebellar Intrinsic Activity Underlying Executive Dysfunction in ASD

PerAF captures fluctuation amplitude of the resting‐state BOLD signals in present units, representing local spontaneous functional activity. In this study, we observed no significant between‐group differences in PerAF, whereas predominantly increased functional interactions within the cerebro‐cerebellar loops were identified in the ASD group compared with the TD group. Our results are in line with a previous study conducted by Wang et al. [37], who reported no significant differences in regional cerebellar activity between ASD and TD individuals, despite evident alterations in cerebro‐cerebellar connectivity. Such a dissociation between stable cerebellar regional activity and altered network‐level connectivity may suggest that cerebellar involvement in ASD may be mediated through the cerebro‐cerebellar interactions, rather than localized alterations in intrinsic cerebellar activity. Nevertheless, it should be noted that the between‐group comparisons of cerebellar PerAF in the present study were restricted to a limited number of hypothesis‐driven ROIs. This analytical strategy may have reduced sensitivity to detect potential regional alterations at the cerebellar level. Thus, conclusions regarding the absence of regional PerAF differences in the selected ROIs in ASD individuals should be interpreted with caution. Future studies employing voxel‐based comparison analysis may provide a more comprehensive view of cerebellar intrinsic activity among ASD individuals.

### 4.2. Altered Cerebro‐Cerebellar Connectivity Associated With BRIEF Difficulties Among ASD

Individuals with ASD showed predominantly increased FC in cerebro‐cerebellar circuits compared with TD participants. Importantly, FC strength between the left Lobule VI and right IFGoper, as well as between the left Crus I and left IFGoper, was positively correlated with the shift subscale in BRIEF. These positive associations suggest that greater FC within these cerebro‐cerebellar loops correlated with poorer executive control.

This pattern aligns with prior reports of cerebro‐cerebellar hyperconnectivity in ASD. For example, Khan et al. [24] found predominant overconnectivity within the cerebro‐cerebellar functional loop among individuals with ASD, suggesting that this overconnectivity may be associated with early white matter overgrowth [38–40]. They further interpreted the difference in FC alteration as a characteristic of child development, where supramodal cognition emerges later than sensorimotor function, resulting in a cerebro‐cerebellar connectivity imbalance [24]. Extending this account, we suggested that hyperconnectivity may reflect maladaptive over‐coupling within these circuits, potentially imposing a biased allocation of neural resources and thereby relating to greater real‐world difficulties in cognitive flexibility and emotional regulation.

The shift subscale of the BRIEF assesses an individual’s ability to flexibly adapt to changing circumstances. Such ability often involves updating contextual information during cognitive control, which engages a distributed functional network including the prefrontal cortex [41]. Using probabilistic reversal‐learning task to evaluate cognitive flexibility, Wang et al. found increased responses in the prefrontal cortex during the switch state. Although IFGoper was classically associated with language processing, converging evidence indicated that IFGoper plays an important role in executive control. For example, lesions involving the IFGoper were associated with prolonged stop‐signal reaction time (SSRT), which measures response inhibition in the stop‐signal task and thereby supports flexible adaptation to changing task demands [42]. Meanwhile, transcranial magnetic stimulation targeting the IFGoper altered SSRT [43]. From an anatomical perspective, IFGoper lies adjacent to the inferior frontal junction (IFJ), which was found consistently engaged during task switching and was considered to contribute to cognitive processes common to attention shifting in a domain‐general manner [44].

### 4.3. Limitation

This study has limitations. First, cerebellar ROIs were predefined based on the previous ALE analysis, which may have overlooked other cerebellar regions that could contribute to EF among ASD individuals. Meanwhile, our cerebellar ROIs were defined using a seed‐based approach. Given evidence that cerebellar lobular boundaries do not necessarily align with functional subdivisions, future studies could apply functional parcellation atlases to further characterize cerebellar functional organization. Moreover, potential site effects arising from differences between the two datasets should be considered, as we did not implement explicit procedures to control for site effects in the present study. Since EF involves multiple cognitive factors that may overlap, future studies should focus on differentiating these cognitive factors and further analyzing the cerebellum’s role in EF with respect to specific cognitive subtypes.

## 5. Conclusion

The present study showed predominantly increased FC within cerebro‐cerebellar circuits in ASD compared with TD. Notably, FC strength between the left Lobule VI and right IFGoper, as well as between the left Crus I and left IFGoper were positively correlated with the shift subscale in BRIEF. These results together suggested the maladaptation to the changing environment of ASD individuals might be in part reflected by alterations in functional interconnection between the cerebellum and the cerebrum. These findings help to understand the potential role of the cerebellum in EF among ASD individuals and might help to provide ideas for therapeutic interventions among individuals with ASD.

## Author Contributions

Xiaotong Zhang, Zhiming Tang, and Zhaowen Zhou conceived and designed the study. Guorong Qiu and Changfu Chen performed data extraction and preprocessing. Xiaotong Zhang and Zhaowen Zhou conducted data analysis, prepared the figures, and wrote the original draft. Zhiwei Mou and Jianliang Lu reviewed and revised the manuscript. Zhiming Tang and Zhaowen Zhou supervised the project and acquired funding.

## Funding

The work was supported by the China Postdoctoral Science Foundation (Grant 2025M781906), the Guangzhou Municipal Basic and Applied Basic Research Program (Grant 2024A04J4181), the Guangdong Provincial Administration of Traditional Chinese Medicine Research Program (Grant 20261076), the Guangdong Basic and Applied Basic Research Foundation (Grant 2024A1515010597), the Scientific and Technological Planning Project of Guangzhou City (Grant 2024B03J1341), the Specialized Strategy for Scientific and Technological Innovation of Guangdong Province (Medical Research and Tackling Key Problems 004), and the Guangdong Health Information Net Association Research Program (Grant HX‐202507‐0006).

## Disclosure

The funding organizations played no further role in study design, data collection, analysis and interpretation, and paper writing.

## Ethics Statement

This study utilized publicly available data from ABIDE II. The ethical statement can be viewed on the ABIDE website https://fcon_1000.projects.nitrc.org/indi/abide/. As introduced by ABIDE II, “prior to data contribution, sites are required to confirm that their local Institutional Review Board (IRB) or ethics committee has approved both the initial data collection and the retrospective sharing of a fully de‐identified version of the datasets (i.e., after removal of the 18 protected health information identifiers, including facial information from structural images, as identified by the Health Insurance Portability and Accountability Act [HIPAA]).”

## Conflicts of Interest

The authors declare no conflicts of interest.

## Supporting Information

Additional supporting information can be found online in the Supporting Information section.

## Supporting information


**Supporting Information** Table S1. Detailed MRI acquisition parameters. Table S2. Clinical characteristics of included ASD individuals for two sites. Table S3. Between‐group difference in seed‐based FC analysis (ASD < TD). Figure S1. Correlation analysis between BRIEF scores and FC related to ROI1. Figure S2. Correlation analysis between BRIEF scores and FC related to ROI2. Figure S3. Correlation analysis between BRIEF scores and FC related to ROI3. Figure S4. Correlation analysis between BRIEF scores and FC related to ROI4.

## Data Availability

The data that support the findings of this study are openly available in ABIDE II at https://fcon_1000.projects.nitrc.org/indi/abide/abide_II.html.
