# Supplementary material for: Executive Dysfunction in Autism Spectrum Disorder Is Associated With Increased Cerebro‐Cerebellar Resting‐State Functional Connectivity
Source: Neural Plast. 2026 Mar 16;2026:7449692. doi: 10.1155/np/7449692 (PMC13140381; doi:10.1155/np/7449692)
Supplement: Supplementary file 1 — Supporting Information Table S1. Detailed MRI acquisition parameters. Table S2. Clinical characteristics of included ASD individuals for two sites. Table S3. Between‐group difference in seed‐based FC analysis (ASD < TD). Figure S1. Correlation analysis between BRIEF scores and FC related to ROI1. Figure S2. Correlation analysis between BRIEF scores and FC related to ROI2. Figure S3. Correlation analysis between BRIEF scores and FC related to ROI3. Figure S4. Correlation analysis between BRIEF scores and FC related to ROI4. [file NP-2026-7449692-s001.docx]

Table S1. Detailed MRI acquisition parameters

|  | KKI dataset | SDSU dataset |
| --- | --- | --- |
| scanner | 3T Philips Achieva scanner (8-channel head coil) | 3T GE MR750 scanner (8-channel head coil) |
| Rs-fMRI EPI |  |  |
| TR/TE | 2500/30 ms | 2000/30 ms |
| Flip angle | 75° | 90° |
| slices | 47 | 42 |
| Voxel size | 3.00 × 3.00 × 3.00 mm | 3.44 × 3.44 × 3.40 mm |
| volumes | 156 volumes with 2 dummy volumes discarded | 180 volumes with 5 initial dummy volumes discarded |

Table S2. Clinical characteristics of included ASD individuals for two sites

|  | KKI dataset  (n=41) | SDSU dataset  (n=30) |
| --- | --- | --- |
| Age | 10.35±1.52 | 13.08±3.29 |
| Sex |  |  |
| Male | 29 (70.73%) | 23 (76.67%) |
| Female | 12 (29.27%) | 7 (23.33%) |
| FIQ | 104.03±17.24 | 100.30±14.55 |
| VIQ | 111.29±18.30 | 98.07±15.41 |
| PIQ | 104.85±15.20 | 102.70±18.32 |
| BRIEF t-score |  |  |
| Inhibit | 63.83±10.96 | 66.43±15.63 |
| Shift | 65.17±9.44 | 74.53±12.37 |
| Emotional control | 57.24±11.01 | 66.27±11.64 |
| Initiate | 62.49±8.75 | 68.07±11.13 |
| Working memory | 67.85±9.56 | 66.90±12.18 |
| Plan/ Organize | 66.29±10.38 | 68.60±9.08 |
| Organization of materials | 60.98±10.52 | 58.57±12.29 |
| Monitor | 65.95±10.72 | 58.57±12.29 |
| BRI | 63.51±8.83 | 71.23±12.78 |
| MI | 67.63±9.25 | 68.07±9.71 |
| GEC | 67.29±8.04 | 70.73±10.78 |

**Table S3.** Between-group difference in seed-based FC analysis (ASD<TD)

|  |  | MNI coordinates | | |  | |
| --- | --- | --- | --- | --- | --- | --- |
|  | Region | x | y | z | Cluster size | Peak-*t* |
| ROI1 | | | | | | |
| A | left Lobule IX | -12 | -39 | -45 | 308 | -6.9396 |
| B | right thalamus | 3 | -12 | 0 | 1458 | -7.4043 |
| C | left fusiform gyrus | -21 | -81 | -6 | 30 | -4.2151 |
| D | left inferior frontal gyrus (pars opercularis) | -36 | 18 | 15 | 68 | -4.7588 |
| E | right inferior frontal gyrus (pars opercularis) | 39 | 18 | 15 | 38 | -4.4017 |
| ROI2 | | | | | | |
| A | right Vermis_10 | 6 | -51 | -27 | 377 | -6.0601 |
| B | right hippocampus | 30 | -39 | 6 | 1449 | -7.384 |
| C | left inferior frontal gyrus (pars opercularis) | -39 | 15 | 15 | 120 | -5.2124 |
| D | right inferior frontal gyrus (pars triangularis) | 45 | 24 | 12 | 143 | -4.8059 |
| ROI3 | | | | | | |
| A | right Vermis_10 | 6 | -51 | -27 | 659 | -6.6994 |
| B | right middle temporal pole | 30 | 12 | -42 | 35 | 3.7896 |
| C | right hippocampus | 24 | -33 | 3 | 7801 | -6.6154 |
| D | right middle frontal gyrus | 36 | 36 | 21 | 31 | -3.3462 |
| E | left medial superior frontal gyrus | 0 | 54 | 33 | 37 | -3.4324 |
| F | left middle frontal gyrus | -30 | 12 | 39 | 31 | -4.2576 |
| G | left superior frontal gyrus | -12 | 6 | 51 | 79 | -4.1773 |
| H | left supplementary motor area | 0 | 9 | 63 | 51 | -3.4455 |
| ROI4 | | | | | | |
| A | right Lobule IX | 3 | -57 | -51 | 67 | -5.2089 |
| B | left Crus II | -21 | -90 | -30 | 36 | -4.5912 |
| C | right Crus I | 33 | -87 | -30 | 63 | -4.7944 |
| D | right hippocampus | 30 | -39 | 6 | 212 | -5.3592 |
| E | left hippocampus | -24 | -30 | -6 | 227 | -5.0143 |
| F | right Heschl gyrus | 42 | -21 | 6 | 318 | -5.5171 |
| G | left inferior frontal gyrus (pars opercularis) | -36 | 15 | 15 | 146 | -5.2866 |
| H | right thalamus | 3 | -18 | 3 | 88 | -6.0136 |
| I | left Rolandic Operculum | -42 | -12 | 12 | 99 | -4.4775 |
| J | right caudate | 9 | 18 | 12 | 51 | -5.4608 |


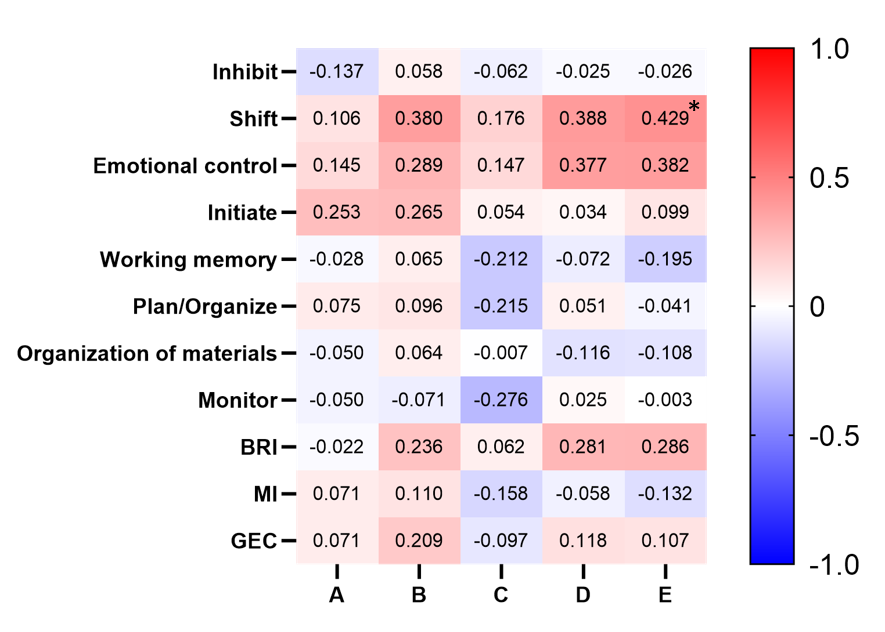


**Figure S1.** Correlation analysis between BRIEF scores and FC related to ROI1

A: FC in left Lobule VI - left Lobule IX; B: FC in left Lobule VI - right thalamus; C: FC in left Lobule VI -left fusiform gyrus; D: FC in left Lobule VI - left inferior frontal gyrus (pars opercularis); E: right inferior frontal gyrus (pars opercularis); BRI: Behavioral Regulation Index; MI: Metacognition Index; GEC: Global Executive Composite; * indicated FDR-adjusted P<0.05.


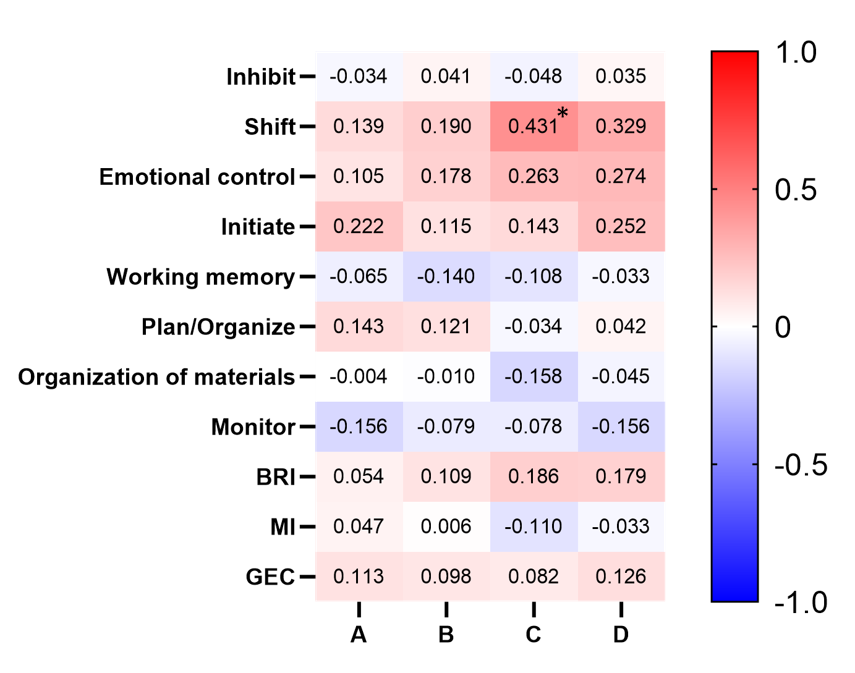


**Figure S2.** Correlation analysis between BRIEF scores and FC related to ROI2

A: FC in left Crus I - right Vermis_10; B: FC in left Crus I -right hippocampus; C: FC in left Crus I -left inferior frontal gyrus (pars opercularis); D: FC in left Crus I - right inferior frontal gyrus (pars triangularis); BRI: Behavioral Regulation Index; MI: Metacognition Index; GEC: Global Executive Composite; * indicated FDR-adjusted P<0.05.


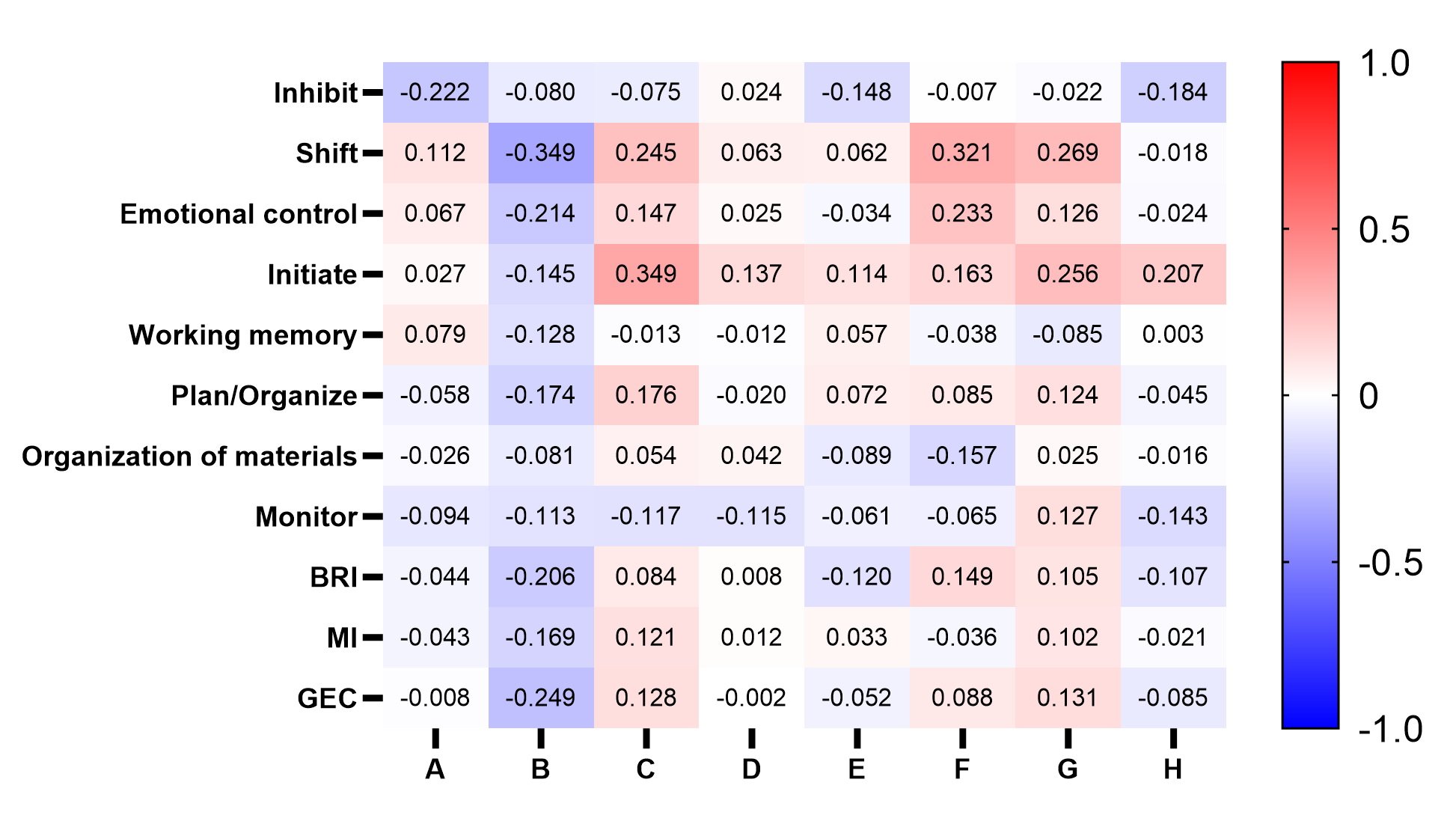


**Figure S3.** Correlation analysis between BRIEF scores and FC related to ROI3

A: FC in right Crus I- right Vermis_10; B: FC in right Crus I- right middle temporal pole; C: FC in right Crus I- right hippocampus; D: FC in right Crus I- right middle frontal gyrus; E: FC in right Crus I- left medial superior frontal gyrus; F: FC in right Crus I- left middle frontal gyrus; G: FC in right Crus I- left superior frontal gyrus; H: FC in right Crus I- left supplementary motor area. BRI: Behavioral Regulation Index; MI: Metacognition Index; GEC: Global Executive Composite


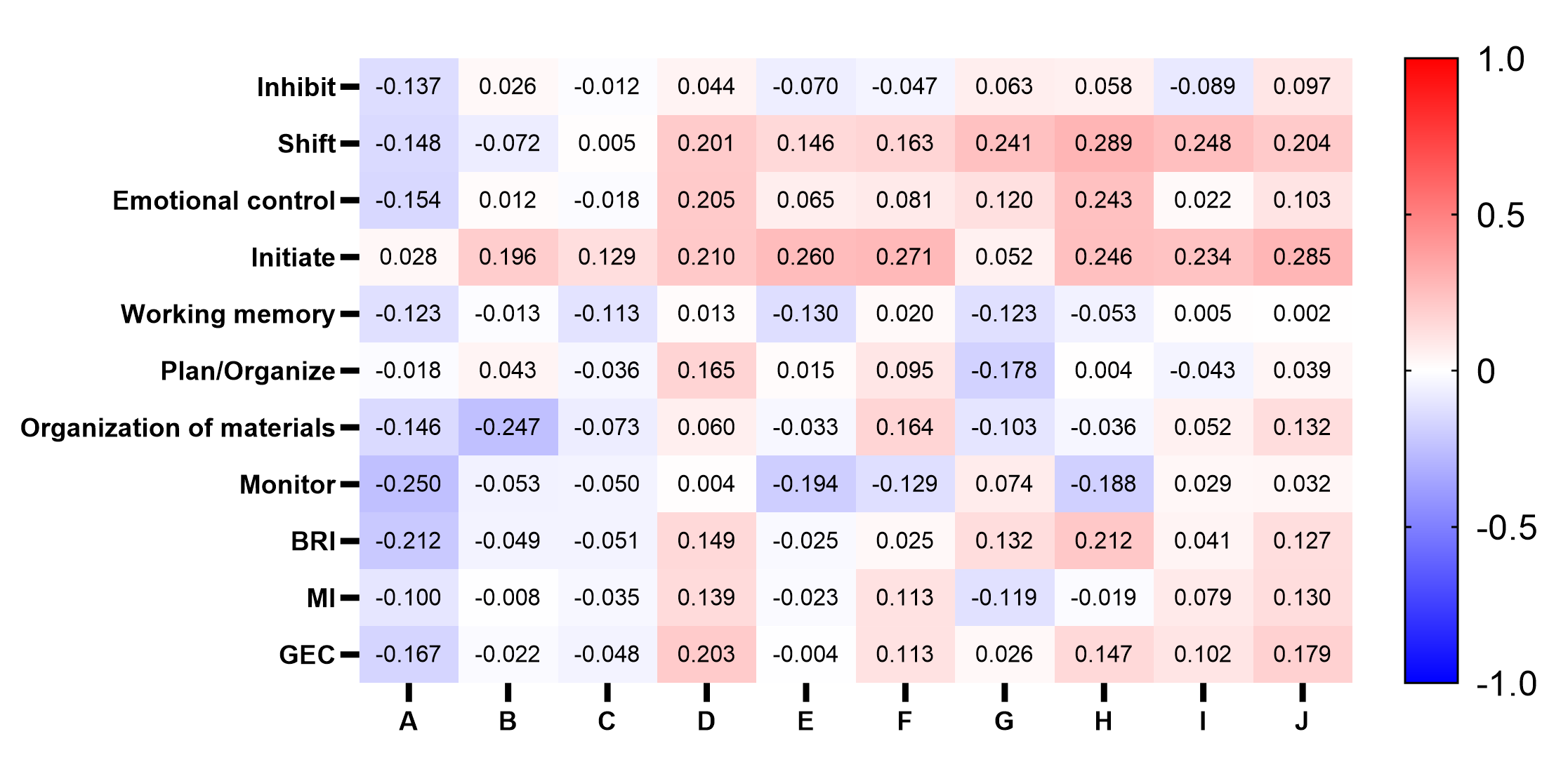


**Figure S4.** Correlation analysis between BRIEF scores and FC related to ROI4

A: FC in left VIIB - right Lobule IX; B: FC in left VIIB - left Crus II; C: FC in left VIIB - right Crus I; D: FC in left VIIB - right hippocampus; E: FC in left VIIB - left hippocampus; F: FC in left VIIB - right Heschl gyrus; G: FC in left VIIB - left inferior frontal gyrus (pars opercularis); H: FC in left VIIB - right thalamus; I: FC in left VIIB - left Rolandic Operculum; J: FC in left VIIB - right caudate; BRI: Behavioral Regulation Index; MI: Metacognition Index; GEC: Global Executive Composite
